# A novel compound heterozygous mutation of *COL6A3* in Chinese patients with isolated cervical dystonia

**DOI:** 10.3389/fneur.2023.1105760

**Published:** 2023-04-04

**Authors:** Rui Wu, Weikang Dou, Huimin Zhou, Ming Shi

**Affiliations:** ^1^Department of Neurology, Xijing Hospital, Fourth Military Medical University, Xi'an, Shaanxi, China; ^2^Department of Neurology, Xi'an People's Hospital (Xi'an Fourth Hospital), Xi'an, Shaanxi, China

**Keywords:** cervical dystonia, *COL6A3*, novel variant, compound heterozygous mutation, whole-exome sequencing

## Abstract

**Background:**

The etiology and pathogenesis of idiopathic dystonia remain obscure. Recent studies revealed that compound heterozygous mutations in collagen type VI alpha-3 gene *COL6A3* may cause recessive isolated dystonia (DYT)-27. However, whether *COL6A3* mutations are associated with Chinese patients with isolated dystonia is not yet reported.

**Methods:**

In this study, 45 Chinese patients with isolated cervical dystonia were recruited, and their blood DNA samples were subjected to whole-exome sequencing. The potential causal variants of *COL6A3* were identified based on the criteria of the American College of Medical Genetics and Genomics and by prediction software.

**Results:**

Among 45 isolated cervical dystonia patients, 18 patients (10 female patients and eight male patients) were found to have seven potential causal variants in the *COL6A3* gene. Among these variants, a compound heterozygous mutation was found in one patient. One allele had a c.1264G>A mutation in exon 4 that resulted in an amino acid substitution of methionine for valine at codon 422 (p.Val422Met) and the other a c.8965+9G>A mutation involving a splicing change in exon 40. In addition, other five missense variants, including c.958G>A (p.Ala320Thr), c.1478T>C (p.Val493Ala), c.1597C>T (p.Arg533Cys), c.1762G>A (p.Asp588Asn), and c.4912G>A (p.Ala1638Thr), were identified as well.

**Conclusion:**

We identified a novel deleterious compound heterozygous mutation as well as five missense variants in the *COL6A3* gene of Chinese patients with cervical dystonia. These findings may expand the spectrum of the *COL6A3* genotype in isolated dystonia.

## Background

Dystonia is a kind of hyperkinetic movement disorder characterized by intermittent or sustained muscle contractions causing involuntary movements and/or abnormal postures in one or more parts of the body (1, 2). At present, the etiology and pathogenesis of dystonia remain largely unclear. The majority view is that dystonia is a neural network disorder, and specific gene variation may be one of the important causes underlying dystonia pathogenesis (3–6). With the rapid development of next-generation sequencing technology, more and more pathogenic genes have been discovered, providing perspectives for our in-depth understanding of dystonia development (7–10).

Recently, the collagen-type VI alpha-3 gene (*COL6A3)* has been reported to be associated with early-onset isolated dystonia (DYT)-27 (11). *COL6A3* encodes the alpha-3 chain of type VI collagen, which is an important component of the extracellular matrix, involving the coordination of synaptogenesis and the stability of the synaptic networks (12). It was reported that compound heterozygous mutations in the *COL6A3* gene may be responsible for (DYT)-27 syndrome (11, 13, 14). However, to the best of our knowledge, at present, only seven cases of compound heterozygous *COL6A3* in dystonia were reported in Caucasian and Indian patients (11, 13, 14). Whether these compound heterozygous mutations or other variants in *COL6A3* were present in Chinese dystonia patients have not been reported so far. Therefore, in this study, we analyzed the mutations of *COL6A3* in 45 Chinese patients with isolated cervical dystonia by next-generation sequencing and tried to identify potential causal variants of *COL6A3*.

## Methods and materials

### Subjects

Overall, 45 unrelated Chinese Han patients (20 male patients and 25 female patients) in middle adulthood were recruited from the movement disorder outpatient clinic of the Department of Neurology of Xijing Hospital, Fourth Military University (Xi'an, China), between April 2020 and June 2022. Trying to avoid the data were not affected by either early development or environment, and the patients with relatively younger ages (under middle adulthood) were recruited. They were diagnosed with isolated cervical dystonia by a specialist in a movement disorder. The patients with combined dystonia or suspected of other acquired etiologies were not included in this study. All the patients had no family history of any type of dystonia, and their family members did not present similar dystonic symptoms as well. The Col-Cap concept was applied to classify the clinical subtypes of cervical dystonia (15).

### Sequencing and genetic analysis

After obtaining the informed consent, we collected patients' peripheral blood samples. After genomic DNAs were extracted, DNA samples were fragmented and then subjected to DNA library creation using established Illumina paired-end protocols. Exome capture was performed by using the SureSelect Human All Exon V6 Kit (Agilent Technologies, Santa Clara, CA, USA) according to the manufacturer's instructions. Genomic DNA sequencing was carried out in the Illumina NovaSeq 6000 platform (Illumina Inc., San Diego, CA, USA). The average sequencing depth was 132.9 ± 15.1 with a depth-of-coverage ≥10 x for at least 99% of the targeted regions. The sequences obtained were aligned to the human reference genome (GRCH37) by using Burrows–Wheeler Aligner (Ver.0.7.8-r455). Single-nucleotide variants (SNVs) and INDELs were identified with SAMtools (Ver. 1.6), and copy number variants were detected by CoNIFER software (Ver. 0.2.2). Acquired variants were annotated by using ANNOVAR (2017 June 8) and a set of disease databases, including the ClinVar database (https://www.ncbi.nlm.nih.gov/clinvar) (2022), the Online Mendelian Inheritance in Man (OMIM) (2022), and the Human Gene Mutation Database (HGMD) (2015). Then, these variants were classified into pathogenic, likely pathogenic, uncertain significance (VUS), likely benign, or benign according to the American College of Medical Genetics and Genomics (ACMG) criteria.

After classification, *COL6A3* mutations with the pathogenic, likely pathogenic, or VUS variants were sorted out, which were used for further harmfulness analysis through filtration by following methods: (1) those with a minor allele frequency (MAF) of <1% in the population databases, including 1000g_Chinese (2015), 1000g_all (2015), esp6500siv2_all (2014), gnomAD_ALL (2017), and gnomAD_EAS (2017), was reserved; (2) only SNVs occurring in exons or exon–intron junctions (≤10 bp) were selected; synonymous SNVs which are not relevant to the amino acid alternation predicted by dbscSNV were discarded; (3) small-fragment non-frameshift (<10 bp) INDELs in the repeat region defined by RepeatMasker were discarded; and (4) the variations were screened according to the scores of SIFT (https://sift.bii.a-star.edu.sg), Polyphen (http://genetics.bwh.harvard.edu/pph2), MutationTaster (http://www.mutationtaster.org), and CADD (http://cadd.gs.washington.edu) software. A CADD score of more than 10 was used as a cutoff, according to previous studies (16, 17). Finally, the potential causal variants were retained if the score from ≥2 software supported their potential harmfulness. Sites (>2 bp) that did not affect the alternative splicing were discarded.

### Sanger sequencing

The variants of *COL6A3* were validated by Sanger sequencing on the ABI 3730xl genetic analyzer (Applied Biosystems, USA). The forward and reverse primers were used for amplifying the *COL6A3* gene (Supplemental Table 1). Sequencing data for sample chromatograms were assessed using Chromas Lite 2.1.1 software.

## Results

### Clinical manifestations

After analyzing the genetic information of 45 Chinese patients with isolated cervical dystonia by whole-exome sequencing, we found that 18 (10 female patients and eight male patients) patients had variants of *COL6A3* (NM_004369.4, OMIM 120250). Their basic information, clinical manifestations, and genetic information are shown in Table 1. The mean age at onset was 41.6 ± 7.3 years ranging from 24 to 52 years. The course of the disease ranged from 2 to 85 months. All the patients had no family history of movement disorder. According to the Col-Cap concept (15), nine patients displayed torticaput, five patients showed torticaput with laterocaput, three patients showed torticaput with retrocaput, and one patient showed laterocaput. In addition, 14 patients were accompanied by other symptoms, such as tremors, pain, and both (Table 1).

**Table 1:** Phenotypic profile of 18 patients with *COL6A3* mutation.

**Patient No**.	**Age**	**Sex**	**Age at onset**	**Course of disease (m)**	**Family history**	**Clinical symptoms**	**Accompanied symptoms**	***COL6A3* mutation**
S4	40	M	38	26	Neg	Torticaput + laterocaput	Tremor and pain	c.1065C>T
S8	38	F	38	6	Neg	Torticaput	Tremor	c.4912G>A
S10	47	F	47	2	Neg	Torticaput	Pain	c.1264G>A; c.8965+9G>A
S12	43	M	43	6	Neg	Torticaput	Tremor and pain	c.1478T>C
S14	40	M	38	18	Neg	Torticaput		c.9148G>A
S19	52	M	51	9	Neg	Laterocaput		c.4614C>T
S21	51	F	48	36	Neg	Torticaput + laterocaput	Tremor	c.1597C>T; c.9148G>A
S23	29	F	27	24	Neg	Torticaput	Pain	c.4912G>A
S24	47	F	39	85	Neg	Torticaput + retrocaput		c.1762G>A
S30	42	F	40	24	Neg	Torticaput + retrocaput	Pain	c.1065C>T; c.1264G>A
S32	39	F	38	8	Neg	Torticaput	Tremor	c.237T>C
S33	24	M	21	36	Neg	Torticaput + laterocaput	Tremor and pain	c.237T>C; c.4900+9C>T
S34	43	F	39	40	Neg	Torticaput	Tremor and pain	c.958G>A
S36	37	F	36	13	Neg	Torticaput	Tremor	c.4912G>A
S39	41	M	40	8	Neg	Torticaput + laterocaput	Tremor and pain	c.4900+9C>T
S40	39	F	39	5	Neg	Torticaput	Tremor	c.4900+9C>T
S42	45	M	43	22	Neg	Torticaput + laterocaput	Pain	c.8097G>A
S44	52	M	51	6	Neg	Torticaput + retrocaput		c.4184G>A

F, female; M, male; m, months; neg, negative.

### Genetic analysis

By whole-exome sequencing, approximately 120,000 deleterious and conserved variants per sample were first obtained. After ACMG classification, approximately 25,000 variants were screened and reserved. Thereafter, we sorted the *COL6A3* gene out and found eight missense variants, four synonymous variants, and two splicing variants (Tables 1, 2). All the variants were classified as VUS (Table 2). Theoretically, it is pathogenic and likely pathogenic and not VUS variants that are believed to cause diseases. However, recent evidence showed that although most VUS variants were reclassified into benign or likely benign, there were still parts of VUS variants that were reclassified into pathogenic or likely pathogenic variants (18). Therefore, to further predict these VUS variants' harmfulness, a series of methods were used as described in the Method section. Through harmful filtration, seven potential harmful variants were the reserves in nine patients (Table 3), namely c.958G>A (p.Ala320Thr), c.1264G>A (p.Val422Met), c.1478T>C (p.Val493Ala), c.1597C>T (p.Arg533Cys), c.1762G>A (p.Asp588Asn), c.4912G>A (p.Ala1638Thr), and c.8965+9G>A (Table 3).

**Table 2:** Information of *COL6A3* variants after ACMG classification.

**Patient No**.	**cDNA**	**Protein**	**Exon**	**SNP ID**	**Het/Hom**	**Mutation type**	**ACMG classification (evidence)**
S32, S33	c.237T>C	p.Ala79=	3	rs747312241	Het	exonic	VUS (1BP + 1PM)
S34	c.958G>A	p.Ala320Thr	4	rs115819851	Het	exonic	VUS (1BP + 1PM)
S4, S30	c.1065C>T	p.Ala355=	4	rs115155458	Het	exonic	VUS (1BP)
S10, S30	c.1264G>A	p.Val422Met	4	rs114511558	Het	exonic	VUS (1BP + 1PP)
S12	c.1478T>C	p.Val493Ala	5	rs116794756	Het	exonic	VUS (1BP + 1PP)
S21	c.1597C>T	p.Arg533Cys	5	rs751952844	Het	exonic	VUS (1PM)
S24	c.1762G>A	p.Asp588Asn	5	rs886043408	Het	exonic	VUS (1PM)
S44	c.4184G>A	p.Arg1395Gln	9	rs80272723	Het	exonic	VUS (1BA + 1BS + 1BP + 1PP)
S19	c.4614C>T	p.Asp1538=	10	rs199759398	Het	exonic	VUS (1BP + 1PM)
S33, S39, S40	c.4900+9C>T		10	rs117345850	Het	splicing	VUS (1BP)
S8, S23, S36	c.4912G>A	p.Ala1638Thr	11	rs114322958	Het	exonic	VUS (1BP + 2PP)
S42	c.8097G>A	p.Val2699=	38	rs115757876	Het	exonic	VUS (1BP)
S10	c.8965+9G>A		40	-	Het	splicing	VUS (1PM)
S14, S21	c.9148G>A	p.Ala3050Thr	41	rs114596320	Het	exonic	VUS (1BP)

ACMG, American College of Medical Genetics and Genomics criteria; BA, stand-alone evidence of benign; BP, supporting evidence of benign; BS, strong evidence of benign; Het, heterozygous; Hom, homozygous, PM, moderate evidence of pathogenicity; PP, supporting evidence of pathogenicity; VUS, variants of uncertain significance.

-, not available.

**Table 3:** Potential causal variants in *COL6A3* after harmful filtration.

**Patient no**.	**cDNA**	**Protein**	**Exon**	**Het/Hom**	**Mutation type**	**1000g_ Chinese**	**1000g_ ALL**	**esp6500si_ all**	**gnomAD_ ALL_AF**	**gnomAD_ EAS_AF**	**SIFT**	**Polyphen2**	**Mutation taster**	**CADD Score^a^**
S34	c.958G > A	p.Ala320Thr	4	Het	exonic	0.004983	0.000799	0.000077	0.000314	0.003977	Tolerate	Possible damage	Disease causing	22.8
S10^*^, S30	c.1264G > A	p.Val422Met	4	Het	exonic	0.013289	0.001997	-	0.000641	0.008818	Deleterious	Probably damage	Disease causing	22.6
S12	c.1478T > C	p.Val493Ala	5	Het	exonic	0.013289	0.001997	0.000077	0.001212	0.016163	Tolerate	Probably damage	Disease causing	21.0
S21	c.1597C > T	p.Arg533Cys	5	Het	exonic	-	-	-	0.000016	-	Deleterious	Probably damage	Polymorphism	20.5
S24	c.1762G > A	p.Asp588Asn	5	Het	exonic	-	-	-	0.000012	-	Deleterious	Probably damage	Polymorphism	22.2
S8, S23, S36	c.4912G > A	p.Ala1638Thr	11	Het	exonic	0.006645	0.000799	-	0.000641	0.006631	Deleterious	Probably damage	Disease causing	25.5
S10^*^	c.8965+9G > A		40	Het	splicing	-	-	-	-	-	-	-	-	12.30

Het, heterozygous; Hom, homozygous; PolyPhen2, polymorphism phenotyping v2; SIFT, sorting intolerant from tolerant.

-, not available.

a CADD: The score more than 10 is considered as deleteriousness for SNP.

* Patient S10 presents two potential deleterious variants in COL6A3.

**Figure 1:**
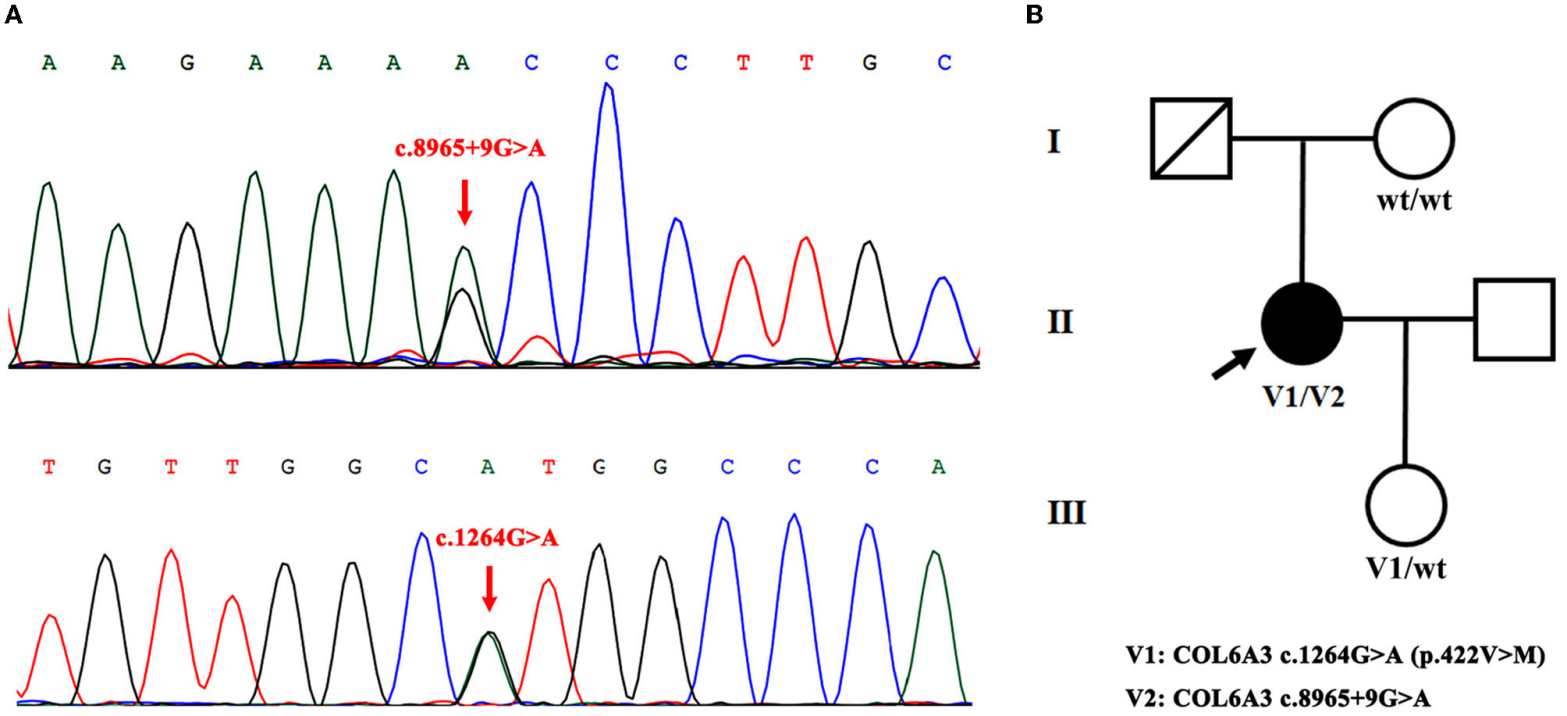
Novel compound heterozygous mutation of *COL6A3* in patient S10. **(A)** Sanger sequencing chromatograms of portions of *COL6A3* gene compound mutation (c.8965+9G>A and c.1264G>A) (red arrowed) in patient S10. **(B)** Family pedigree chart of patient S10 (V1/V2). The patient's father has passed away, her mother did not have either of the two variants (wt/wt), and her daughter had the mutation of c.1264G>A (V1/wt).

Among these variants, a novel compound heterozygous mutation of c.1264G>A and c.8965+9G>A was found in patient S10. Specifically, c.1264G>A (rs114511558) was a missense change in exon 4 that resulted in an amino acid substitution of methionine for valine at codon 422 (p.Val422Met) (Table 2). By searching population databases, c.1264G>A was found to be a rare variant in 1000g_ALL (MAF = 0.001997), gnomAD_ALL (MAF = 0.000641), and gnomAD_EAS (MAF = 0.008818). It was predicted to be damaging by SIFT, PolyPhen2, and MutationTaster. The CADD score was 22.6 (Table 3). For c.8965+9G>A (Chr2: 238244769), it was a splicing change in exon 40 and not recorded in dbSNP (Table 2), 1000g_Chinese, 1000g_all, esp6500siv2, gnomAD_ALL, and gnomAD_EAS. The CADD score was 12.3 (Table 3). This compound heterozygous mutation was verified by Sanger sequencing (Figure 1A), and the pedigree analysis showed that the patient's father has been dead and her mother did not have either of the two variants, but her daughter had the mutation of c.1264G>A (Figure 1B, Supplemental Figure 1). Except the proband, all family members did not display any types of dystonia (Figure 1B).

In addition, other three compound heterozygous mutations were also found in patients S21 (c.1597C>T and c.9148G>A), S30 (c.1065C>T and c.1264G>A), and S33 (c.237T>C and c.4900+9C>T) (Table 1). All these variants were classified as VUS (Table 2). However, through harmful filtration, we found that only one variation (c.1597C>T in patient S21 and c.1264G>A in patient S30) was harmful (Table 3), and the other (c.9148G>A and c.1065C>T) was harmless. In patient S33, both c.237T>C and c.4900+9C>T were of undetermined significance (data not shown). Thus, we considered that these three compound heterozygous mutations may be not disease-causing.

In addition to compound heterozygous mutations, we also identified five missense variants in *COL6A3*, namely c.958G>A (p.Ala320Thr), c.1478T>C (p.Val493Ala), c.1597C>T (p.Arg533Cys), c.1762G>A (p.Asp588Asn), and c.4912G>A (p.Ala1638Thr). Detailed information on these variants is shown in Table 3. It was noted that c.4912G>A (p.Ala1638Thr and rs114322958) was found in patients S8, S23, and S36 simultaneously. In addition to these harmful sites, other seven VUS variants were also identified, including four synonymous variants [c.237T>C (p.Ala79=), c.1065C>T (p.Ala355=), c.4614C>T (p.Asp1538=), and c.8097G>A (p.Val2699=)], two missense variants [c.4184G>A (p.Arg1395Gln) and c.9148G>A (p.Ala3050Thr)], and one splicing variant (c.4900+9C>T). Intriguingly, some variants were present in different patients simultaneously. For instance, c.237T>C (p.Ala79=) was present in patients S32 and S33, c.1065C>T (p.Ala355=) in patients S4 and S30, c.9148G>A (p.Ala3050Thr) in patients S14 and S21, and c.4900+9C>T in patients S33, S39, and S40 (Table 2).

## Discussion

Mutations in the *COL6A3* gene cause autosomal-recessive early-onset isolated dystonia, namely (DYT)-27, with interindividual heterogeneity of focal, segmental, or generalized distribution in the cranio-cervical region, upper limbs, and trunk (19). Recent evidence showed that the compound heterozygous mutations of *COL6A3* may be disease-causing (11, 13, 14). Here, we examined genetic information on the *COL6A3* gene in 45 Chinese patients with isolated cervical dystonia and found that 18 patients had seven potential causal variants in the *COL6A3* gene. Importantly, among these variants, a novel compound heterozygous mutation of *COL6A3* (c.1264G>A and c.8965+9G>A) was identified.

The *COL6A3* gene encodes the collagen alpha-3 chain, which is one of the three subunits (COL6α1, COL6α2, and COL6α3) of collagen type VI, a microfibrillar component of the extracellular matrix (20, 21). Collagen VI dysfunctions are known to cause two main types of muscle disorders: Ullrich congenital muscular dystrophy and Bethlem myopathy (22, 23). Zech et al. first reported that *COL6A3* mutation was associated with autosomal-recessive (DYT)-27 (11), in which they identified disease-segregating compound heterozygous mutations of *COL6A3* in five cases affected by isolated dystonia from three unrelated German families, specifically, two siblings with c.9128G>A (p.Arg3043His) and c.9245C>G (p.Pro3082Arg), two siblings with c.7502G>A (p.Arg2501His) and c.8966-1G>C (p.Val2989_Lys3077delinsGlu), and one patient from the other family with c.7660G>A (p.Ala2554Thr) and c.8966-1G>C (p.Val2989_Lys3077delinsGlu). Intriguingly, they found that all affected individuals had at least one pathogenic allele in exon 41, promoting them to postulate that exon 41 may be a hot spot for mutations causing isolated dystonia (11).

In our study, by analyzing the *COL6A3* gene by whole-exome sequencing in 45 Chinese patients with isolated cervical dystonia, we found four compound heterozygous mutations (Table 1) and further identified a novel deleterious mutation in patient S10 after harmful filtration, that is, c.1264G>A (p.Val422Met) in exon 4 and c.8965+9G>A in exon 40. This patient was a 47-year-old woman with a complete presentation of cervical dystonia phenotype, consistent with (DYT)-27 manifestation (19). Since in the family pedigree, the patient's father had passed away and her mother did not have either of the two variants while her daughter only had the mutation of c.1264G>A (Figure 1, Supplemental Figure 1), we supposed that c.1264G>A may be from the father and c.8965+9G>A may be *de novo*. Admittedly, for lacking the father's genetic information, we still did not rule out the possibility that other factors, such as environmental modifiers (e.g., perinatal adversities, drug abuse, infections, general anesthesia, or physical trauma) (6, 24) and other variants in different genes, could co-segregate with identified compound heterozygous mutations and contribute to dystonia pathogenesis. In addition, it is noted that both variants were not in exon 41 that was inconsistent with Zech et al.'s report. We supposed that different ethnicities between German and Chinese might be the responsible factor for the absence of exon 41 mutation in our case. At this point, Panda et al. also identified a pathogenic compound heterozygous mutation, not in exon 41 but in exon 10 [c.7557C>T (p.Gly1517Ser)] and exon 12 [c.4498G>A (p.Pro1894Leu)] of the *COL6A3* gene in an Indian case with early-onset isolated dystonia (14). Moreover, although Lohmann et al. found that one German patient carried a compound heterozygous mutation, one in exon 41 [c.9245C>G (p.Pro3082Arg)] and the other in exon 6 [c.2195C>T (p.Thr732Met)] of the *COL6A3* gene when examining 955 patients with isolated or combined dystonia or with another movement disorder with dystonic features, this patient was diagnosed as parkinsonism with dystonic posturing due to homozygous *PINK1* mutations (13). In our study, both patients S14 and S21 had a missense VUS variant [c.9148G>A (p.Ala3050Thr)] in exon 41 (Table 2), but this variant was filtered as harmlessness (Table 3). Thus, we assume that mutations in exon 41 may be just one of the causes of isolated dystonia. Moreover, as a fact, only a few cases of compound heterozygous *COL6A3* in dystonia were reported to date (11, 13, 14), and more new cases in the future should be reported to demonstrate the relationship between compound heterozygous *COL6A3* and the occurrence of dystonia.

In addition to compound heterozygous mutations, we also identified other five missense variants in the *COL6A3* gene, including c.958G>A (p.Ala320Thr), c.1478T>C (p.Val493Ala), c.1597C>T (p.Arg533Cys), c.1762G>A (p.Asp588Asn), and c.4912G>A (p.Ala1638Thr). It is especially noteworthy that patients S8, S23, and S36 carried c.4912G>A (p.Ala1638Thr) simultaneously (Table 3). In addition, several filtered VUS variants should deserve our attention as well (Table 2), though some of them were synonymous mutations, which, however, are reported as harmful as the non-synonymous mutations that alter proteins (25). For instance, c.4900+9C>T was simultaneously present in patients S33, S39, and S40; c.1065C>T (p.Ala355=) was present in patients S4 and S30; and c.237T>C (p.Ala79=) was present in patients S32 and S33. Therefore, these variants might also have the possibility of contributing to dystonia pathogenesis. Admittedly, whether these variants can really cause isolated dystonia needs further studies to be clarified.

Collagen VI represents a remarkable extracellular matrix molecule known for its roles in muscle and connective tissue. In addition to these, it also functions in the coordination of synaptogenesis and the stability of the synaptic networks (12). Zech M et al. revealed that *COL6A3* was expressed in neurons, and the suppression of the exon 41 ortholog caused deficits in an axonal outgrowth without overt collagen defects probably because the exon 41 encodes part of the collagen VI α3 C4 domain (FN-III motif), which might be involved in the organization of structural plasticity (11). Apart from the C4 domain, the von Willebrand factor type-A (vWFA) domains have been shown to bind extracellular matrix proteins, cell-to-cell interaction, and other signaling pathways (14, 22). In Panda et al.'s study, both variants (Gly1517Ser and Pro1894Leu) were found to locate in the vWFA domain of the COL6α3 protein, which was supposed to be involved in other functions in the brain extracellular matrix such as neuronal organization, plasticity, and neuronal circuit formation (14). In our study, for the novel deleterious compound heterozygous mutation, the missense variant c.1264G>A (p.Val422Met) lies in exon 4, located in the vWFA domain of the COL6α3 protein, and the splicing variant c.8965+9G>A located in the downstream of exon 40, probably affecting the C4 domain just as variant c.8966-1G>C located in the upstream of exon 41 reported previously (11).

In conclusion, we identified a novel deleterious compound heterozygous mutation in the *COL6A3* gene in Chinese patients with cervical dystonia. To the best of our knowledge, this study is the fourth report [Zech et al. (11); Lohmann et al. (13); Panda et al. (14)] on the compound heterozygous mutation of *COL6A3* for the dystonia onset. Therefore, our findings may expand the spectrum of the *COL6A3* genotype in the development of isolated dystonia.

## Data availability statement

The datasets presented in this study can be found in online repositories. The name of the repository and accession number can be found below: National Center for Biotechnology Information (NCBI) ClinVar, https://www.ncbi.nlm.nih.gov/clinvar, SCV002553216, SCV002553244 - SCV002553248, SCV002558734, SCV002558735, and SCV002586278 - SCV002586283.

## Ethics statement

The study was approved by the Medical Ethics Committee of the Xijing Hospital. The patients/participants provided their written informed consent to participate in this study. Written informed consent was obtained from the individual(s) for the publication of any potentially identifiable images or data included in this article.

## Author contributions

MS and RW conceived the idea and drafted the manuscript. MS, RW, WD, and HZ contributed to the collection and interpretation of the data. All authors participated in the revision of the manuscript and figure and read and approved the final manuscript.

## Supplementary material

Supplemental Table 1 Primer sequences for amplifying the *COL6A3* gene (NM_004369.4).

Supplemental Figure 1 Sanger sequencing of *COL6A3* gene in patient S10's family. The chromatograms of portions of *COL6A3* gene in the patient's mother **(A)** and daughter **(B)**. Her daughter had the mutation of c.1264G>A (red arrowed).
