# Supplementary material for: A novel compound heterozygous mutation of COL6A3 in Chinese patients with isolated cervical dystonia
Source: Front Neurol. 2023 Apr 4;14:1105760. doi: 10.3389/fneur.2023.1105760 (PMC10110855; doi:10.3389/fneur.2023.1105760)
Supplement: Supplemental Table 1 — Primer sequences for amplifying the COL6A3 gene (NM_004369.4). [file Table_1.DOCX]

**Supplemental table 1** Primer sequences for amplifying the *COL6A3* gene ((NM_004369.4))

| Exon | SNP ID | Variants | Forward primer | Reverse primer |
| --- | --- | --- | --- | --- |
| 3 | rs747312241 | c.237T>C | TAACACTACGATAACCTGAGGGACT | TGCATTGATAGGAAGTAATTGCTTA |
| 4 | rs115819851 | c.958G>A | GGACAGTAAACACCAAGTTGTCATC | CATTATTTTCCTTATTGATGGATCA |
| 4 | rs115155458 | c.1065C>T | GATACATACCTTGTGTGACAATGGT | AGTCATTCTCGACTTCCTTGTAA |
| 4 | rs114511558 | c.1264G>A | ATGCTGGTACCCACCTTACG | GGCTAGCGTGTTCTCATTCG |
| 5 | rs116794756 | c.1478T>C | GAACTCGTGAATAGGTTGTTACGAA | GAGGGTGTCCTTTAAATGGAGTTAG |
| 5 | rs751952844 | c.1597C>T | CTGCTGTTTTCATCATTTGTTGTCT | TCAACAAGAGAGACATAGTCTTCCT |
| 5 | rs886043408 | c.1762G>A | TGTTTTCATCATTTGTTGTCTCTTA | TGCTCTAGACTTTGTTCGTAACAAC |
| 9 | rs80272723 | c.4184G>A | TGAACCTGTAGTCCTAGCTACTGAG | TCATCTCGTCTGGAAAGTCTGA |
| 10 | rs199759398 | c.4614C>T | AAATGTTGATGTCACACTCTGTAGT | TTTTGTTAGCAGGATTGTTCGA |
| 10 | rs117345850 | c.4900+9C>T | AGTGATGGAAGATCAATAATATGCT | GCAAGAAACCTCTTTGTTAAGT |
| 11 | rs114322958 | c.4912G>A | TACTGGACAAGCCCCACTTG | GGCATGGATTTGAGTGAAGG |
| 38 | rs115757876 | c.8097G>A | CTTTTCCATGACTGTTCCTACACTT | AGAAGTACATAGCGTACCTGGTCAG |
| 40 | - | c.8965+9G>A | TGGAATCCCTAACCAACACC | GTAGCAGCAAAGCCAGCAG |
| 41 | rs114596320 | c.9148G>A | AGTAAGTGTCTCCTTTGTGTCCTAT | GATCTATCTTTCAGTTAAGATGTCC |

-, not available.
