# Supplementary figures and images for: A novel compound heterozygous mutation of COL6A3 in Chinese patients with isolated cervical dystonia
Source: Front Neurol. 2023 Apr 4;14:1105760. doi: 10.3389/fneur.2023.1105760 (PMC10110855; doi:10.3389/fneur.2023.1105760)

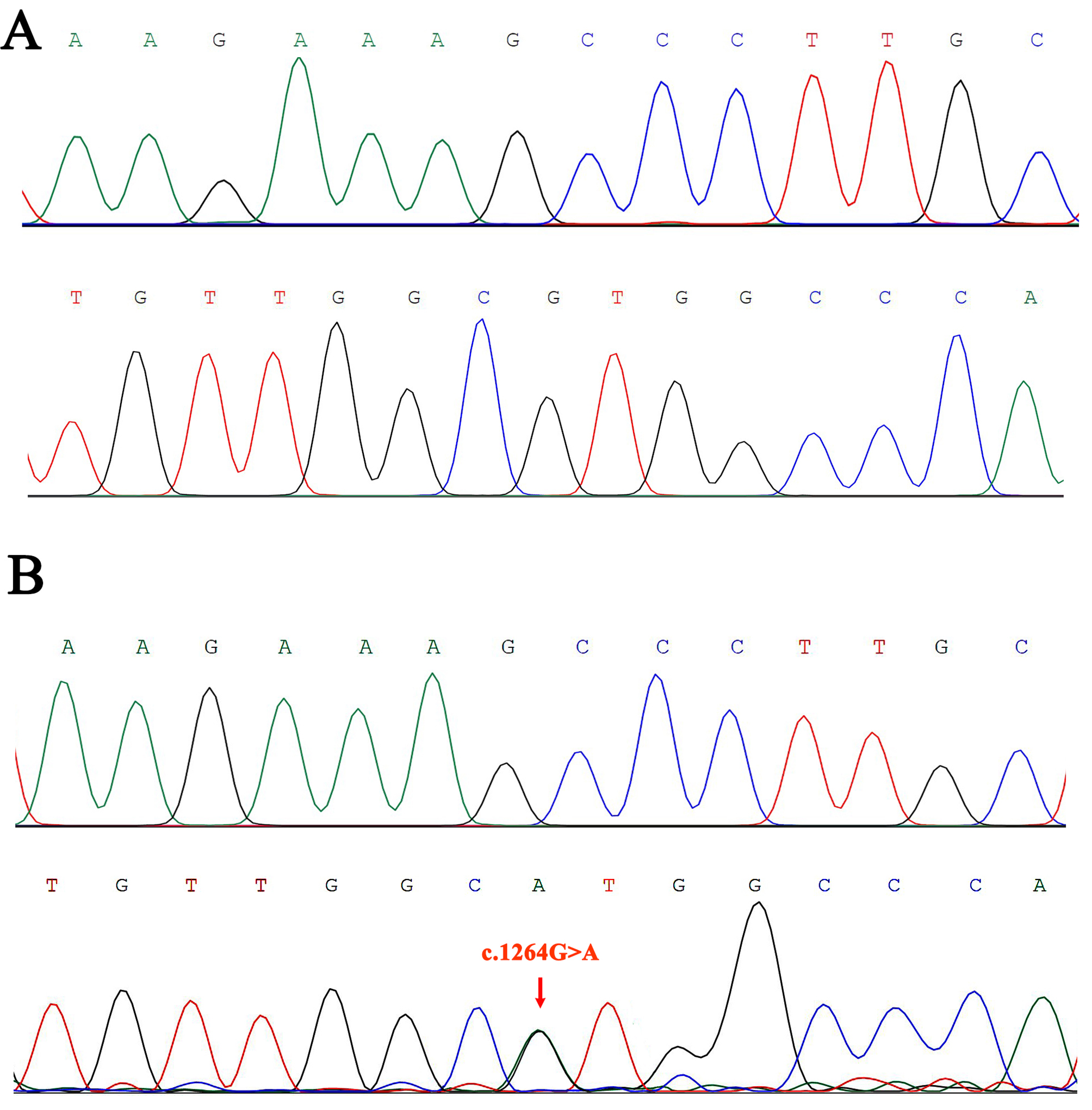

Supplement: Supplemental Figure 1 — Sanger sequencing of COL6A3 gene in patient S10's family. The chromatograms of portions of COL6A3 gene in the patient's mother (A) and daughter (B). Her daughter had the mutation of c.1264G>A (red arrowed). [file Image_1.JPEG]
